# Augmenting K-Means Clustering With Qualitative Data to Discover the Engagement Patterns of Older Adults With Multimorbidity When Using Digital Health Technologies: Proof-of-Concept Trial

**DOI:** 10.2196/46287

**Published:** 2024-03-28

**Authors:** Yiyang Sheng, Raymond Bond, Rajesh Jaiswal, John Dinsmore, Julie Doyle

**Affiliations:** 1 NetwellCASALA Dundalk Institution of Technology Dundalk Ireland; 2 School of Computing Ulster University Jordanstown United Kingdom; 3 School of Enterprise Computing and Digital Transformation Technological University Dublin Dublin Ireland; 4 Trinity Centre for Practice and Healthcare Innovation School of Nursing and Midwifery Trinity College Dublin Dublin Ireland

**Keywords:** aging, digital health, multimorbidity, chronic disease, engagement, k-means clustering

## Abstract

**Background:**

Multiple chronic conditions (multimorbidity) are becoming more prevalent among aging populations. Digital health technologies have the potential to assist in the self-management of multimorbidity, improving the awareness and monitoring of health and well-being, supporting a better understanding of the disease, and encouraging behavior change.

**Objective:**

The aim of this study was to analyze how 60 older adults (mean age 74, SD 6.4; range 65-92 years) with multimorbidity engaged with digital symptom and well-being monitoring when using a digital health platform over a period of approximately 12 months.

**Methods:**

Principal component analysis and clustering analysis were used to group participants based on their levels of engagement, and the data analysis focused on characteristics (eg, age, sex, and chronic health conditions), engagement outcomes, and symptom outcomes of the different clusters that were discovered.

**Results:**

Three clusters were identified: the typical user group, the least engaged user group, and the highly engaged user group. Our findings show that age, sex, and the types of chronic health conditions do not influence engagement. The 3 primary factors influencing engagement were whether the same device was used to submit different health and well-being parameters, the number of manual operations required to take a reading, and the daily routine of the participants. The findings also indicate that higher levels of engagement may improve the participants’ outcomes (eg, reduce symptom exacerbation and increase physical activity).

**Conclusions:**

The findings indicate potential factors that influence older adult engagement with digital health technologies for home-based multimorbidity self-management. The least engaged user groups showed decreased health and well-being outcomes related to multimorbidity self-management. Addressing the factors highlighted in this study in the design and implementation of home-based digital health technologies may improve symptom management and physical activity outcomes for older adults self-managing multimorbidity.

## Introduction

### Background

According to the United Nations, the number of people aged ≥65 years is growing faster than all other age groups [1]. The worldwide population of people aged ≥65 years will increase from approximately 550 million in 2000 to 973 million in 2030 [2]. Furthermore, by 2050, approximately 16% of the world’s population will be aged >65 years, whereas 426 million people will be aged >80 years [1]. Living longer is a great benefit to today’s society. However, this comes with several challenges. Aging can be associated with many health problems, including multimorbidity (ie, the presence of ≥2 chronic conditions) [3]. The prevalence rate of multimorbidity among older adults is estimated to be between 55% and 98%, and the factors associated with multimorbidity are older age, female sex, and low socioeconomic status [4]. In the United States, almost 75% of older adults have multimorbidity [5], and it was estimated that 50 million people in the European Union were living with multimorbidity in 2015 [6]. Likewise, the prevalence rate of multimorbidity is 69.3% among older adults in China [5].

Home-based self-management for chronic health conditions involves actions and behaviors that protect and promote good health care practices comprising the management of physical, emotional, and social care [7]. Engaging in self-management can help older adults understand and manage their health conditions, prevent illness, and promote wellness [7,8]. However, self-management for older adults with multimorbidity is a long-term, complex, and challenging mission [9,10]. There are numerous self-care tasks to engage in, which can be very complicated, especially for people with multiple chronic health conditions. Furthermore, the severity of the disease can negatively impact a person’s ability to engage in self-management [10].

Digital home-based health technologies have the potential to support better engagement with self-management interventions, such as the monitoring of symptom and well-being parameters as well as medication adherence [10,11]. Such technologies can help older adults understand their disease or diseases, respond to changes, and communicate with health care providers [12-14]. Furthermore, digital health technologies can be tailored to individual motivations and personal needs [13], which can improve sustained use [15] and result in people feeling supported [16]. Digital self-management can also create better opportunities for adoption and adherence in the long term compared with paper booklet self-management [16]. Moreover, digital health technologies, such as small wearable monitoring devices, can increase the frequency of symptom monitoring for patients with minimal stress compared with symptom monitoring with manual notifications [17].

A large body of research implements data mining and machine learning algorithms using data acquired from home-based health care data sets. Data mining techniques, such as data visualization, clustering, classification, and prediction, to name a few, can help researchers understand users, behaviors, and health care phenomena by identifying novel, interesting patterns. These techniques can also be used to build predictive models [18-21]. In addition, data mining techniques can help in designing health care management systems and tracking the state of a person’s chronic disease, resulting in appropriate interventions and a reduction in hospital admissions [18,22]. Vast amounts of data can be generated when users interact with digital health technologies, which provides an opportunity to understand chronic illnesses as well as elucidate how users engage with digital health technologies in the real world. Armstrong et al [23] used the k-means algorithm to identify previously unknown patterns of clinical characteristics in home care rehabilitation services. The authors used k-means cluster analysis to analyze data from 150,253 clients and discovered new insights into the clients’ characteristics and their needs, which led to more appropriate rehabilitation services for home care clients. Madigan and Curet [22] used classification and regression trees to investigate a home-based health care data set that comprised 580 patients who had 3 specific conditions: chronic obstructive pulmonary disease (COPD), heart failure (HF), and hip replacement. They found that data mining methods identified the dependencies and interactions that influence the results, thereby improving the accuracy of risk adjustment methods and establishing practical benchmarks [22]. Other research [24] has developed a flow diagram of a proposed platform by using machine learning methods to analyze multiple health care data sets, including medical images as well as diagnostic and voice records. The authors believe that the system could help people in resource-limited areas, which have lower ratios of physicians and hospitals, to diagnose diseases such as breast cancer, heart disease (HD), diabetes, and liver disease at a lower cost and in less time than local hospitals. In the study, the accuracy of disease detection was >95% [24].

There are many different approaches to clustering analysis of health care data sets, such as k-means, density-based spatial clustering of applications with noise, agglomerative hierarchical clustering, self-organizing maps, partitioning around medoids algorithm, hybrid hierarchical clustering, and so on [25-28]. K-means clustering is 1 of the most commonly used clustering or unsupervised machine learning algorithms [19,29], and it is relatively easy to implement and relatively fast [30-32]. In addition, k-means has been used in research studies related to chronic health conditions such as diabetes [33], COPD [34,35], and HF [36]; for example, a cloud-based framework with k-means clustering technique has been used for the diagnosis of diabetes and was found to be more efficient and suitable for handling extensive data sets in cloud computing platforms than hierarchical clustering [32]. Violán et al [37] analyzed data from 408,994 patients aged 45 to 64 years with multimorbidity using k-means clustering to ascertain multimorbidity patterns. The authors stratified the k-means clustering analysis by sex, and 6 multimorbidity patterns were found for each sex. They also suggest that clusters identified by multimorbidity patterns obtained using nonhierarchical clustering analysis (eg, k-means and k-medoids) are more consistent with clinical practice [37].

The majority of data mining studies on chronic health conditions focus on the diseases themselves and their symptoms; there is less exploration of the patterns of engagement of persons with multimorbidity with digital health technologies. However, data mining and machine learning are excellent ways to understand users’ engagement patterns with digital health technologies. A study by McCauley et al [38] compared clustering analysis of the user interaction event log data from a reminiscence mobile app that was designed for people living with dementia. In addition to performing quantitative user interaction log analysis, the authors also gathered data on the qualitative experience of users. The study showed the benefits of using data mining to analyze the user log data with complementary qualitative data analysis [38]. This is a research challenge where both quantitative and qualitative methods can be combined to fully understand users; for example, the quantitative analysis of the user event data can tell us about use patterns, the preferred times of day to use the app, the feature use, and so on, but qualitative data (eg, user interviews) are necessary to understand *why* these use patterns exist.

### Objectives

The aim of this study was to analyze how older adults with multimorbidity engage with digital symptom and health monitoring over a period of approximately 12 months using a digital health platform. In this study, user log data of engagement with digital health technology and user interview qualitative data were examined to explore the patterns of engagement. K-means clustering was used to analyze the user log data. The study had four research questions: (1) How do clusters differ in terms of participant characteristics such as age, sex, and health conditions? (2) How do clusters differ in terms of patterns of engagement, such as the number of days a week participants take readings (eg, weight and blood pressure [BP])? (3) How do engagement rates with the different devices correlate with each other (determined by analyzing the weekly submissions of every parameter and the interviews of participants)? and (4) How do engagement rates affect participants’ health condition symptoms, such as BP, blood glucose (BG) level, weight, peripheral oxygen saturation (SpO_2_) level, and physical activity (PA)?

## Methods

### Overview

The study was a proof-of-concept trial with an action research design and mixed methods approach. Action research is a period of investigation that “describes, interprets, and explains social situations while executing a change intervention aimed at improvement and involvement” [39]. An action research approach supports the generation of solutions to practical problems while using methods to understand the contexts of care as well as the needs and experiences of participants.

### Recruitment and Sample

Although 120 participants consented to take part across Ireland and Belgium, this paper reports on data from 60 Irish older adults with multiple chronic health conditions (≥2 of the following: COPD, HF, HD, and diabetes). Participants were recruited through purposive sampling and from multiple sources, including through health care organizations (general practitioner clinics and specialist clinics), relevant older adult networks, chronic disease support groups, social media, and local newspaper advertising. Recruitment strategies included the use of study flyers and advertisements as well as giving talks and platform demonstrations.

### Sources of Data

The data set was collected during the Integrated Technology Systems for Proactive Patient Centred Care (ProACT) project proof-of-concept trial. As the trial was a proof-of-concept of a novel digital health platform, the main goal was to understand how the platform worked or did not work, rather than whether it worked. Thus, to determine sample size, a pragmatic approach was taken in line with two important factors: (1) Is the sample size large enough to provide a reliable analysis of the ecosystem? and (2) Is the sample size small enough to be financially feasible? The literature suggests that overall sample size in proof-of-concept digital health trials is low. A review of 1030 studies on technical interventions for management of chronic disease that focused on HF (436 studies), stroke (422 studies), and COPD (172 studies) suggested that robust sample sizes were 17 for COPD, 19 for HF, and 21 for stroke [40]. Full details on the study protocol can be found in the study by Dinsmore et al [41].

Participants used a suite of sensor devices (ie, BP monitors, weight scales, glucometers, pulse oximeters, and activity watches) and a tablet app to monitor their health conditions and well-being. All participants received a smartwatch to measure PA levels and sleep, a BP monitor to measure BP and pulse rate, and a weight scale. A BG meter was provided to participants with diabetes, and a pulse oximeter was provided to those with COPD to measure SpO_2_ levels. In addition, all participants received an iPad with a custom-designed app, the ProACT CareApp, that allowed users to view their data, provide self-report (SR) data on symptoms that could not be easily captured through a sensor (eg, breathlessness and edema) and well-being (eg, mood and satisfaction with social life), receive targeted education based on their current health status, set PA goals, and share their data with others. The ProACT platform was designed and developed following an extensive user-centered design process. This involved interviews, focus groups, co-design sessions (hands-on design activities with participants), and usability testing before the platform’s deployment in the trial. A total of 58 people with multimorbidity and 106 care network participants, including informal carers, formal carers, and health care professionals, took part in this process. Findings from the user-centered design process have been published elsewhere [42,43]. More detailed information about the full ProACT platform and the CareApp used by participants can be found in the study by Doyle et al [44].

The study took place between April 1, 2018, and June 30, 2019. Participants in the trial typically participated for 12 months, although some stayed on for 14 months and others for 9 months (in the case of those who entered the trial later). One of the trial objectives was to understand real-world engagement. Therefore, participants were asked to take readings with the devices and provide SR data in the ProACT CareApp whenever they wished (not necessarily daily). As part of the trial, participants were assisted by technical help desk staff who responded to questions about the technology, and home visits were conducted as needed to resolve issues. In addition, a clinical triage service monitored the participants’ readings and contacted them in instances of abnormal parameter values (eg, high BP and low SpO_2_ levels) [45]. Participants also received a monthly check-in telephone call from 1 of the triage nurses.

Table 1 outlines the types of health and well-being metrics that were collected, as well as the collection method and the number of participants who collected that type of data. The health and well-being metrics were determined from the interviews and focus groups held with health care professionals during the design of the ProACT platform to determine the most important symptom and well-being parameters to monitor across the health conditions of interest [42]. Off-the-shelf digital devices manufactured by 2 providers, Withings and iHealth, were used during the trial. Data from these providers were extracted into a custom platform called Context-Aware Broker and Inference Engine–Subject Information Management System (CABIE-SIMS), which includes a data aggregator for storing health and well-being data. All devices require the user to interact with them in some way. However, some devices needed more interaction than others (eg, taking a BG reading involved several steps, but PA and sleep only required participants to open the activity watch app to sync the relevant data). The activity watch was supposed to synchronize automatically without user interaction. However, inconsistencies with syncing meant that users were advised to open the Withings app to sync their data. The CABIE-SIMS platform would display the readings in *near* real time, apart from PA data, which were collected at regular intervals throughout the day, whereas sleep data were gathered every morning. Table 1 lists the types of data that were collected and the number of participants who collected them. In addition, semistructured interviews were conducted with all participants at 4 time points throughout the trial to understand their experience of using the ProACT platform. Although a full qualitative thematic analysis was outside the scope of this study and was reported on elsewhere [44], interview transcripts for participants of interest to the analysis presented in this paper were reviewed as part of this study to provide an enhanced understanding of the results.

**Table 1:** Types of data, collection methods, and number of participants collecting these data (n=60).

Data type	Collection method	Participants (at start of trial), n (%)
Blood pressure	Place device on arm and turn on device, which opens Withings Health Mate app to collect data; press “Start” in app to take reading	60 (100)
Pulse rate	Collected as part of blood pressure measurement	60 (100)
Blood glucose level	Turn on device and open app; prepare lancing device by inserting new lancet and setting puncture depth; wash hands thoroughly; insert test strip into device; take blood sample from the finger; apply blood sample to test strip, and wait for result to display; discard test strip and lancet	34 (57)
SpO_2_^a^ level	Place device in current orientation on index finger; turn on device and open app to take reading	22 (37)
Weight	Stand on weight scales; reading is automatically transferred via Wi-Fi to app	60 (100) as lifestyle parameter, including 11 (18) as symptom parameter for HF^b^
Physical activity	Participants advised to open Withings Health Mate app at least once per day to ensure syncing of data	60 (100)
Sleep	Participants advised to open Withings Health Mate app at least once per day to ensure syncing of data	60 (100)
Self-report (general well-being, eg, mood, anxiety, satisfaction, and medication adherence)	Answered through ProACT^c^ CareApp and automatically pulled into the CABIE-SIMS^d^ platform; most questions delivered daily	60 (100)
Self-report (COPD^e^ symptoms, eg, breathlessness and sputum)	Answered through ProACT CareApp and automatically pulled into the CABIE-SIMS platform; questions delivered daily	22 (37)
Self-report (HF symptoms, eg, swelling and nighttime breathlessness)	Answered through ProACT CareApp and automatically pulled into the CABIE-SIMS platform; questions delivered daily	10 (17)

^a^SpO_2_: peripheral oxygen saturation.

^b^HF: heart failure.

^c^ProACT: Integrated Technology Systems for Proactive Patient Centred Care.

^d^CABIE-SIMS: Context-Aware Broker and Inference Engine–Subject Information Management System.

^e^COPD: chronic obstructive pulmonary disease.

### Data Analysis Methods

The original data set in the CABIE-SIMS platform was formatted using the JSON format. As a first step, a JSON-to-CSV file converter was used to make the data set more accessible for data analysis. The main focus was on dealing with duplicate data and missing data during the data cleaning phase. Data duplication might occur when a user uploads their SpO_2_ reading 3 times in 2 minutes as a result of *mispressing* the button. In such cases, only 1 record was added to the cleaned data file. As for missing data, the data set file comprised “N/A” (not available) values for all missing data.

The cleaned data set was preprocessed using Microsoft Excel, the R programming language (R Foundation for Statistical Computing), and RStudio (Posit Software, PBC). The preprocessed data set included participants’ details (ID, sex, age, and chronic health conditions) and the number of days of weekly submissions of every parameter (BP, pulse rate, SpO_2_ level, BG level, weight, PA, SR data, and sleep). All analyses (including correlation analysis, principal component analysis [PCA], k-means clustering, 2-tailed *t* test, and 1-way ANOVA) were implemented in the R programming language and RStudio.

After performing Shapiro-Wilk normality tests on the data submitted each week, we found that the data were not normally distributed. Therefore, Spearman correlation was used to check the correlation among the parameters. Correlation analysis and PCA were used to determine which portions of the data would be included in the k-means clustering. Correlation analysis determined which characteristics or parameters should be selected, and PCA determined the number of dimensions that should be selected as features for clustering. In the clustering process, the weekly submission of each parameter was considered as an independent variable for the discovery of participant clusters, and the outcome of the clustering was a categorical taxonomy that was used to label the 3 discovered clusters. Similarly, the Shapiro-Wilk test was conducted to check the normality of the variables in each group. It was found that most of the variables in each group were normally distributed, and only the weight data submission records of cluster 3, the PA data submission records of cluster 2, the SR data submission records of cluster 3, and the sleep data submission records of cluster 1 were not normally distributed. Therefore, the 2-tailed *t* test and 1-way ANOVA were used to compare different groups of variables. The 2-tailed *t* test was used to compare 2 groups of variables, whereas 1-way ANOVA was used to compare ≥2 groups of variables. *P* values >.05 indicated that there were no statistically significant differences among the groups of variables [46].

As for the qualitative data from the interviews, we performed keyword searches after a review of the entire interview; for example, when the data analysis was related to BP and weight monitoring, a search with the keywords “blood pressure,” “weight,” or “scale” was performed to identify relevant information. In addition, when the aim was to understand the impact of digital health care technology, we focused on specific questions in the second interview, such as “Has it had any impact on the management of your health?”

### Ethical Considerations

Ethics approval was received from 3 ethics committees: the Health Service Executive North East Area Research Ethics Committee, the School of Health and Science Research Ethics Committee at Dundalk Institute of Technology, and the Faculty of Health Sciences Research Ethics Committee at Trinity College Dublin. All procedures were in line with the European Union’s General Data Protection Regulation for research projects, with the platform and trial methods and procedures undergoing data protection impact assessments. Written informed consent was obtained on an individual basis from participants in accordance with legal and ethics guidelines after a careful explanation of the study and the provision of patient information and informed consent forms in plain language. All participants were informed of their right to withdraw from the study at any time without having to provide a reason. Participants were not compensated for their time. Data stored within the CABIE-SIMS platform were identifiable because they were shared (with the participant’s consent) with the clinical triage teams and health care professionals. This was clearly outlined in the participant information leaflet and consent form. However, the data set that was extracted for the purpose of the analysis presented in this paper was pseudonymized.

## Results

### Participants

A total of 60 older adults were enrolled in the study. The average age of participants was 74 (SD 6.4; range 65-92) years; 60% (36) were male individuals, and 40% (24/60) were female individuals. The most common combination of health conditions was diabetes and HD (30/60, 50%), which was followed by COPD and HD (16/60, 27%); HF and HD (7/60, 12%); diabetes and COPD (3/60, 5%); diabetes and HF (1/60, 2%); COPD and HF (1/60, 2%); HF, HD, and COPD (1/60, 2%); and COPD, HD, and diabetes (1/60, 2%). Of the 60 participants, 11 (18%) had HF, 55 (92%) had HD, 22 (37%) had COPD, and 31 (52%) had diabetes. Over the course of the trial, of the 60 participants, 8 (13%) withdrew, and 3 (5%) died. However, this study included data from all participants in the beginning, as long as the participant had at least 1 piece of data. Hence, of the 60 participants, we included 56 (93%) in our analysis, whereas 4 (7%) were excluded because no data were recorded.

### Correlation of Submission Parameters

**Figure 1:**
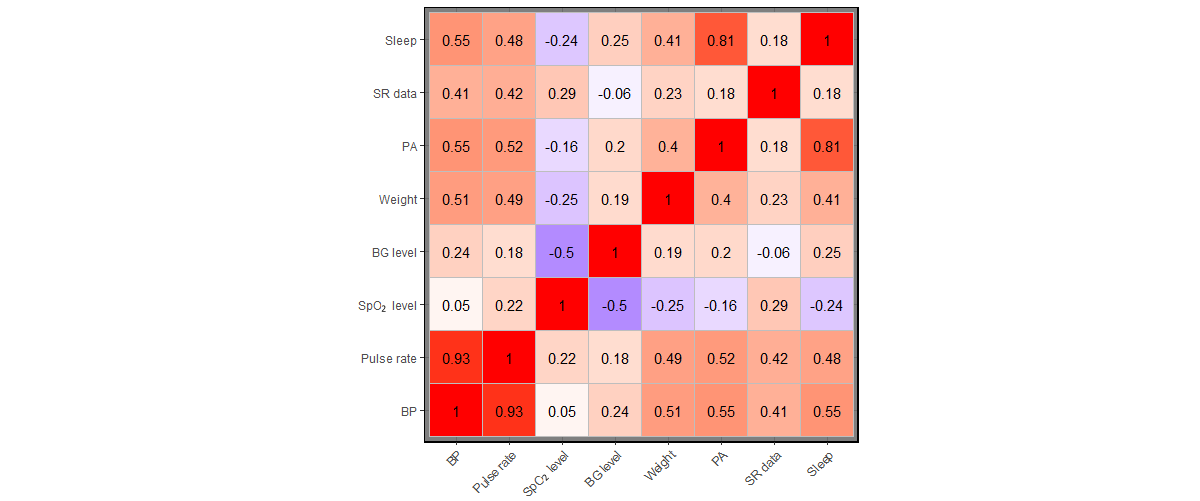
Correlation matrix for weekly submissions (days) of all parameters. BG: blood glucose; BP: blood pressure; PA: physical activity; SpO2: peripheral oxygen saturation; SR: self-report.

To help determine which *distinct* use characteristics or parameters (such as the weekly frequency of BP data submissions) should be selected as features for clustering, the correlations among the parameters were calculated. Figure 1 shows the correlation matrix for all parameter weekly submissions (days). In this study, a moderate correlation (correlation coefficient between 0.3 to 0.7 and −0.7 to −0.3) [47,48] was chosen as the standard for selecting parameters. First, every participant received a BP monitor to measure BP, and pulse rate was collected as part of the BP measurement. Moreover, the correlation coefficient between BP and pulse rate was 0.93, a strong correlation. In this case, BP was selected for clustering rather than pulse rate. As for the other parameters, the correlations between BP and weight (0.51), PA (0.55), SR data (0.41), and sleep (0.55) were moderate, whereas the correlations between BP and SpO_2_ level (0.05) and BG (0.24) were weak. In addition, the correlations between SpO_2_ level and weight (−0.25), PA (0.16), SR data (0.29), and sleep (−0.24) were weak. Therefore, SpO_2_ level was not selected for clustering. Likewise, the correlations between BG and weight (0.19), PA (0.2), SR data (−0.06), and sleep (0.25) were weak. Therefore, BG was not selected for clustering. Thus, BP, weight, PA, SR data, and sleep were selected for clustering.

### PCA and Clustering

**Figure 2:**
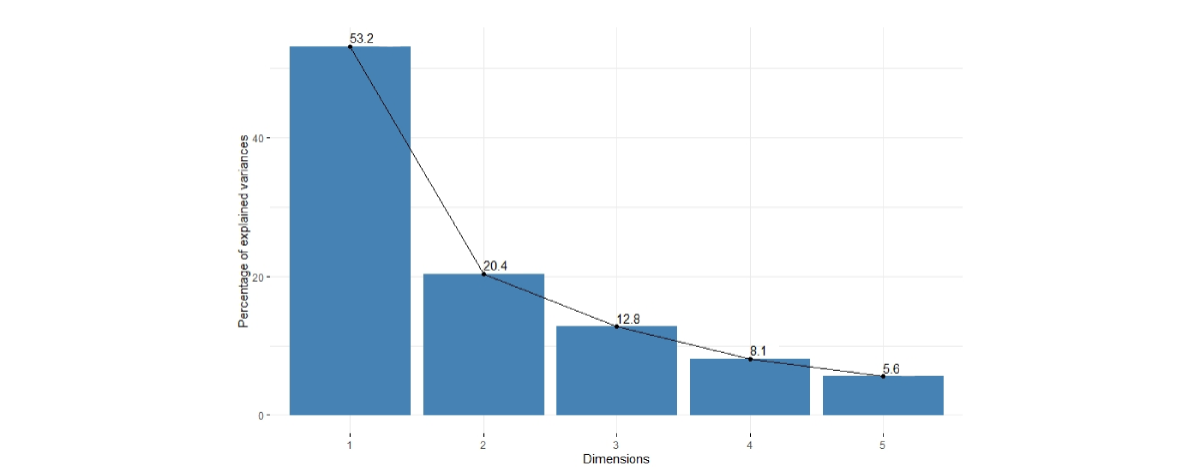
The scree plot of every dimension by principal component analysis.

The fundamental question for k-means clustering is this: how many clusters (k) should be discovered? To determine the optimum number of clusters, we further investigated the data through visualization offered by PCA. As can be seen from Figure 2, the first 2 principal components (PCs) explain 73.6% of the variation, which is an acceptably large percentage. However, after a check of individual contributions, we found that there were 3 participants—P038, P016, and P015—who contributed substantially to PC1 and PC2. After a check of the original data set, we found that P038 submitted symptom parameters only on 1 day, and P016 submitted symptom parameters only on 2 days. Conversely, P015 submitted parameters almost every day during the trial. Therefore, P038 and P016 were omitted from clustering.

**Figure 3:**
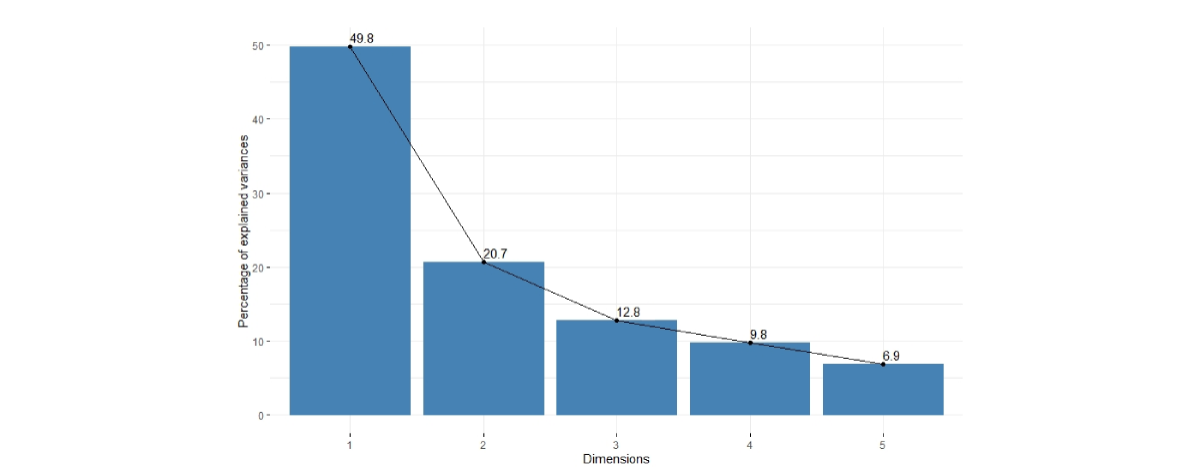
The scree plot of every dimension by principal component analysis (without the outliers).

After removing the outliers (P038 and P016), we found that the first 2 PCs explain 70.5% of the variation (Figure 3), which is an acceptably large percentage.

**Figure 4:**
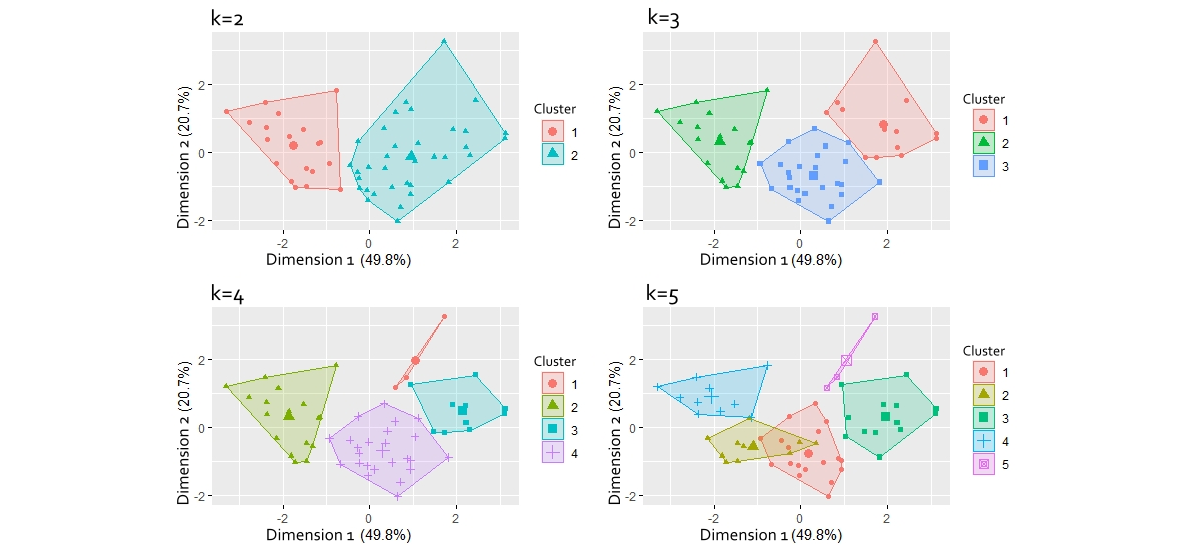
The visualization of clustering with the number of clusters (k) ranging from 2 to 5.

**Figure 5:**
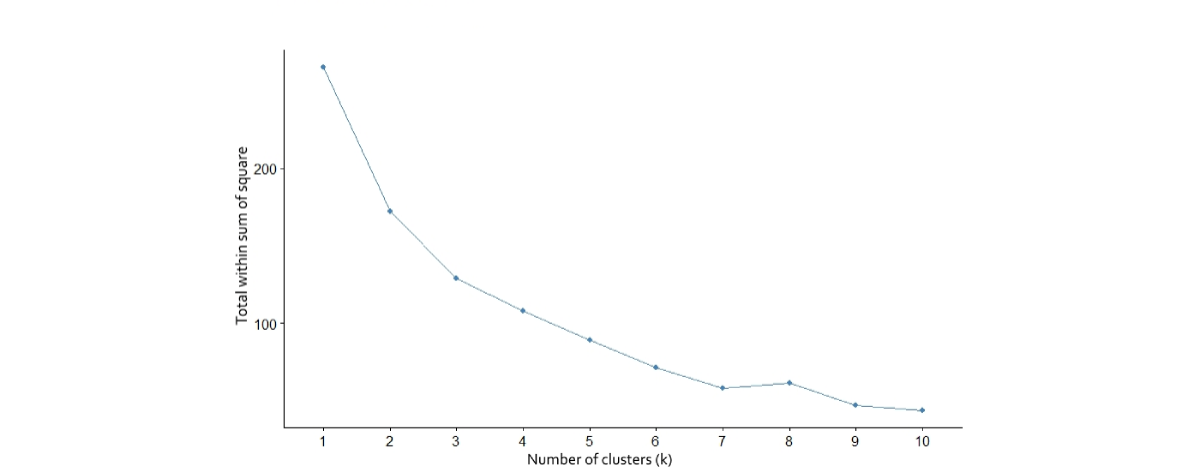
The optimal number of clusters by the elbow method.

The clusters were projected into 2 dimensions as shown in Figure 4. Each subpart in Figure 4 shows a different number of clusters (k). When k=2, the data are obviously separated into 2 big clusters. Similarly, when k=3, the clusters are still separated very well into 3 clusters. When k=4, the clusters are well separated, but compared with the subpart with 3 clusters, 2 clusters are similar, whereas cluster 1, which only has 3 participants, is a relatively small cluster. When k=5, there is some overlap between cluster 1 and cluster 2. Likewise, Figure 5 shows the optimal number of clusters using the elbow method. In view of this, we determined that 3 clusters of participants separate the data set best. The 3 clusters can be labeled as the least engaged user group (cluster 1), the highly engaged user group (cluster 2), and the typical user group (cluster 3).

In the remainder of this section, we report on the examination of the clusters with respect to participant characteristics and the weekly submissions (days) of different parameters in a visual manner to reveal potential correlations and insights. Finally, we report on the examination of the correlations among all parameters by PCA.

### Participant Characteristics

**Figure 6:**
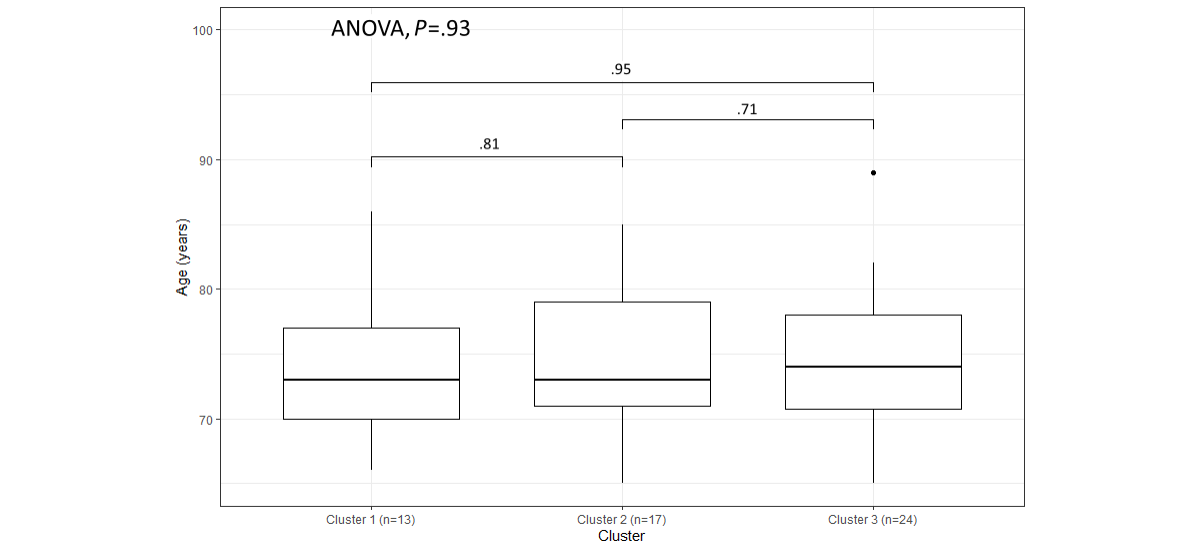
The variation in age within the 3 clusters based on the weekly submissions.

**Figure 7:**
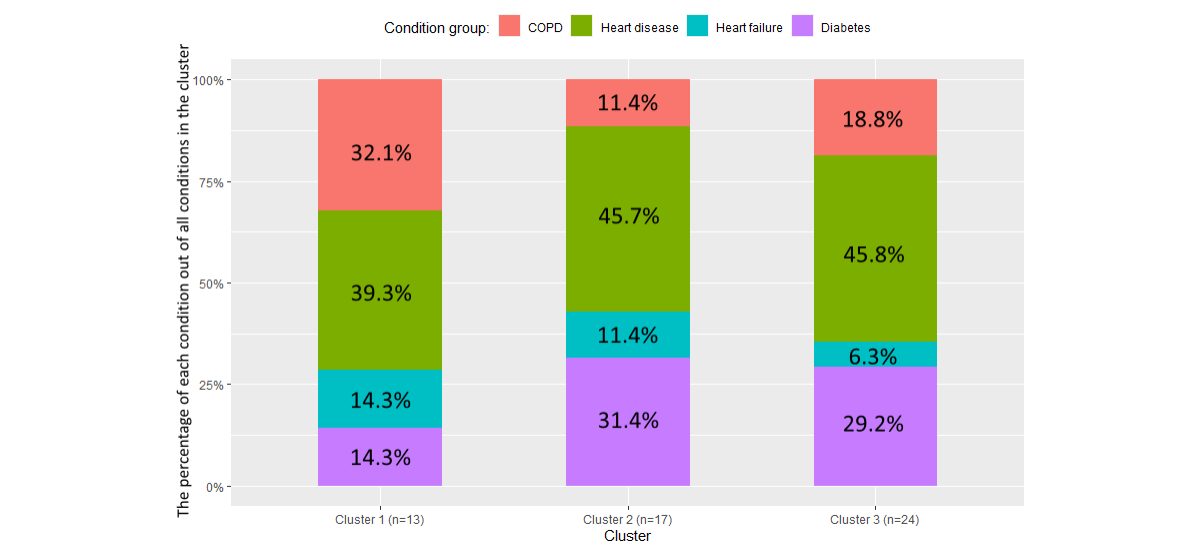
The variation in chronic health conditions within the 3 clusters. Each bar presents the percentage of each condition out of all conditions in the cluster (bearing in mind that participants can have multiple conditions); for example, there are 13 participants and 28 records under the 4 condition groups in cluster 1. Hence, chronic obstructive pulmonary disease (COPD) represents 32.1% of the conditions in cluster 1; however, of the 13 participants in cluster 1, a total of 9 (69%) have COPD, as presented in Table 2.

As seen in Figure 6, the distribution of age within the 3 clusters is similar, with the *P* value of the 1-way ANOVA being .93, because all participants in this trial were older adults. However, the median age in the cluster 3 box plot is slightly higher than the median ages in the box plots of the other 2 clusters, and the average age of cluster 2 participants (74.1 years) is lower than that of cluster 1 (74.6 years) and cluster 3 (74.8 years; Table 2) participants. As Table 2 shows, 6 (26%) of the 23 female participants are in cluster 1 compared with 7 (23%) of the 31 male participants. However, the male participants in cluster 2 (10/31, 32%) and cluster 3 (14/31, 45%) represent higher proportions of total male participants compared with female participants in cluster 2 (7/23, 30%) and cluster 3 (10/23, 43%). Figure 7 shows the proportion of the 4 chronic health conditions within the 3 clusters. Cluster 1 has the largest proportion of participants with COPD and the smallest proportion of participants with diabetes. Moreover, cluster 3 has the smallest proportion of participants with HF (3/24, 13%; Table 2).

**Table 2:** Characteristics of the participants in each cluster (n=54).

Characteristics			Cluster 1 (n=13)		Cluster 2 (n=17)		Cluster 3 (n=24)
Age (y), mean (SD; range)			74.6 (6.2; 66-86)		74.1 (5.5; 65-85)		74.8 (5.9; 65-89)
**Sex, n (%)**							
	Male	7 (23)		10 (32)		14 (45)	
	Female	6 (26)		7 (30)		10 (43)	
**Chronic health conditions, n (%)**							
	COPD^a^	9 (69)		4 (24)		9 (38)	
	Heart disease	11 (85)		16 (94)		22 (92)	
	HF^b^	4 (31)		4 (24)		3 (13)	
	Diabetes	4 (31)		11 (65)		14 (58)	

^a^COPD: chronic obstructive pulmonary disease.

^b^HF: heart failure.

### Participant Engagement Outcomes

**Figure 8:**
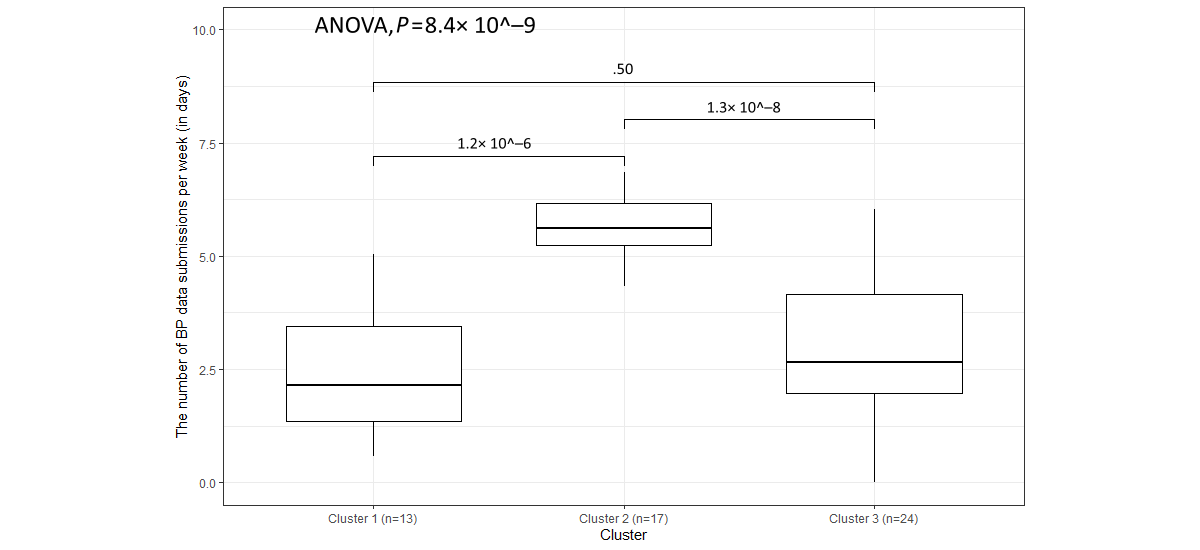
The variation in weekly submissions (days) for blood pressure (BP) data within the 3 clusters.

Cluster 2 has the longest average enrollment time at 352 days compared with cluster 3 at 335 days and cluster 1 at 330 days. As seen in Figure 8, the overall distribution of the BP data weekly submissions is different, with the *P* value of the 1-way ANOVA being 8.4 × 10^−9^. The frequency of BP data weekly submissions (days) of cluster 2 exceeds the frequencies of cluster 1 and cluster 3, which means that participants in cluster 2 have a higher frequency of BP data submissions than those in the other 2 clusters. The median and maximum of cluster 3 are higher than those of cluster 1, but the minimum of cluster 3 is lower than that of cluster 1. Likewise, as seen in Table 3, the mean and SD of cluster 1 (mean 2.5, SD 1.4) are smaller than those of cluster 3 (mean 2.9, SD 2.9).

**Figure 9:**
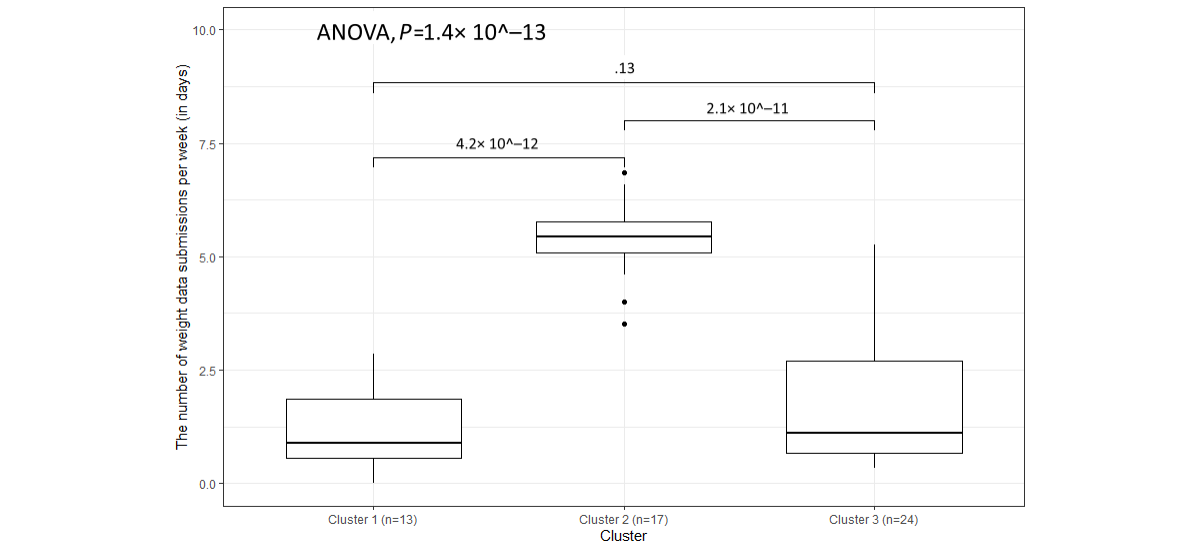
The variation in weekly submissions (days) for weight data within the 3 clusters.

As Figure 9 shows, the overall distribution of the weekly submissions of weight data is different, with the *P* value of the 1-way ANOVA being 1.4 × 10^−13^, because the participants in cluster 2 submitted weight parameters more frequently than those in cluster 1 and cluster 3. In addition, similar to the BP data submissions, the median of cluster 3 is higher than that of cluster 1. As seen in Figure 9, there are 3 outliers in cluster 2. The top outlier is P015, who submitted a weight reading almost every day. During the trial, this participant mentioned many times in the interviews that his goal was to lose weight and that he used the scale to check his progress:

I’ve set out to reduce my weight. The doctor has been saying to me you know there’s where you are and you should be over here. So, I’ve been using the weighing thing just to clock, to track reduction of weight.

P015

The other 2 outliers are P051 and P053, both of whom mentioned taking their weight measurements as part of their daily routine:

Once I get up in the morning the first thing is I weigh myself. That is, the day starts off with the weight, right.

P053

Although their frequency of weekly weight data submissions is lower than that of all other participants in cluster 2, it is still higher than that of most of the participants in the other 2 clusters.

**Figure 10:**
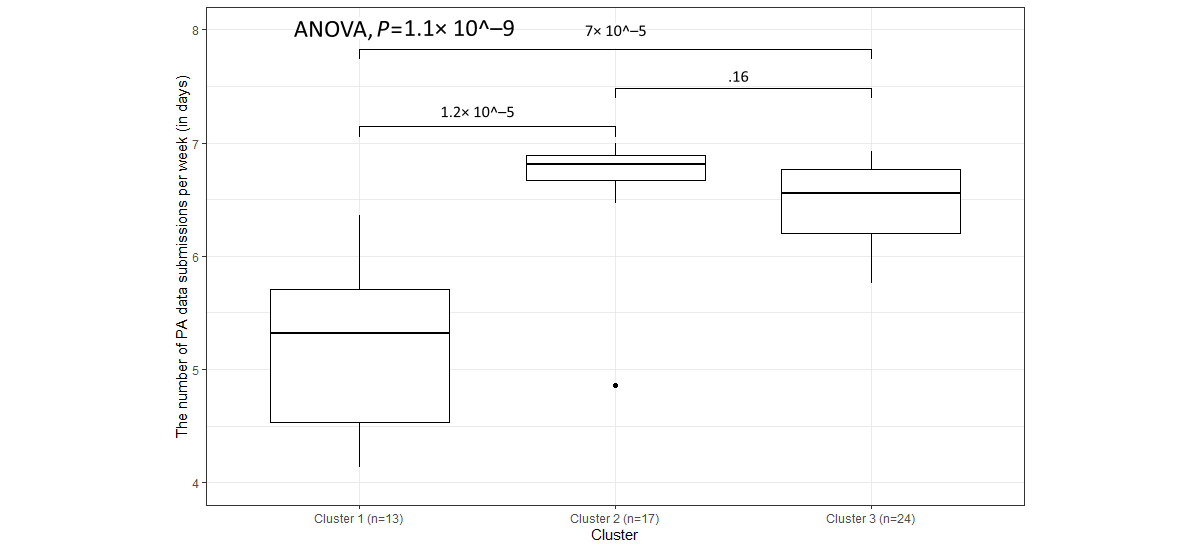
The variation in weekly submissions (days) for physical activity (PA) data within the 3 clusters.

**Figure 11:**
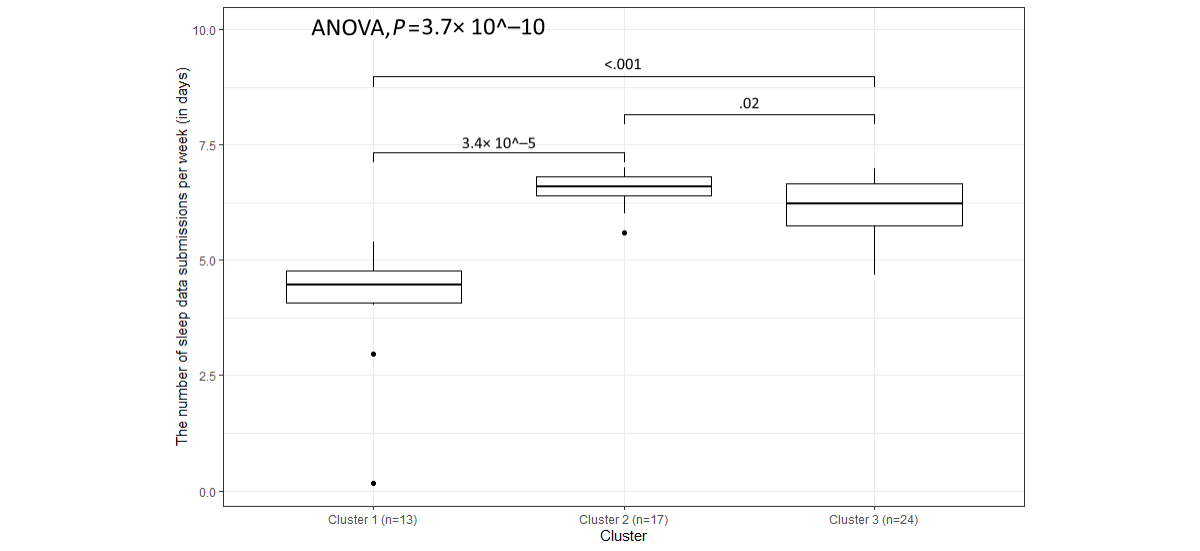
The variation in weekly submissions (days) for sleep data within the 3 clusters.

In Table 3, it can be observed that the average frequency of weekly submissions of PA and sleep data for every cluster is higher than the frequencies of other variables, and the SDs are relatively low. This is likely because participants only needed to open the Withings app once a day to ensure the syncing of data. However, the overall distributions of PA and sleep data submissions are different in Figure 10 and Figure 11, with the *P* values of the 1-way ANOVA being 1.1 × 10^−9^ and 3.7 × 10^−10^, respectively. Moreover, as Figure 10 and Figure 11 show, there are still some outliers who have a low frequency of submissions, and the box plot of cluster 1 is lower than the box plots of cluster 2 and cluster 3 in both figures. The reasons for the low frequency of submissions can mostly be explained by (1) technical issues, including internet connection issues, devices not syncing, and devices needing to be paired again; (2) participants forgetting to put the watch back on after taking it off; and (3) participants stopping using the devices (eg, some participants do not like wearing the watch while sleeping or when they go on holiday):

I was without my watch there for the last month or 3 or 4 weeks [owing to technical issues], and I missed it very badly because everything I look at the watch to tell the time, I was looking at my steps.

P042

I don’t wear it, I told them I wouldn’t wear the watch at night, I don’t like it.

P030

Unlike in the case of other variables, the submission of SR data through the ProACT CareApp required participants to reflect on each question and their status before selecting the appropriate answer. Participants had different questions to answer based on their health conditions; for example, participants with HF and COPD were asked to answer symptom-related questions, whereas those with diabetes were not. All participants were presented with general well-being and mood questions. Therefore, for some participants, self-reporting could possibly take more time than using the health monitoring devices. As shown in Table 3, the frequency of average weekly submissions of SR data within the 3 clusters is relatively small and the SDs are large, which means that the frequency of SR data submissions is lower than that of other variables. Furthermore, there were approximately 5 questions asked daily about general well-being, and some participants would skip the questions if they thought the question was unnecessary or not relevant:

Researcher: And do you answer your daily questions? P027: Yeah, once a week.

Researcher: Once a week, okay. P027: But they’re the same.

**Figure 12:**
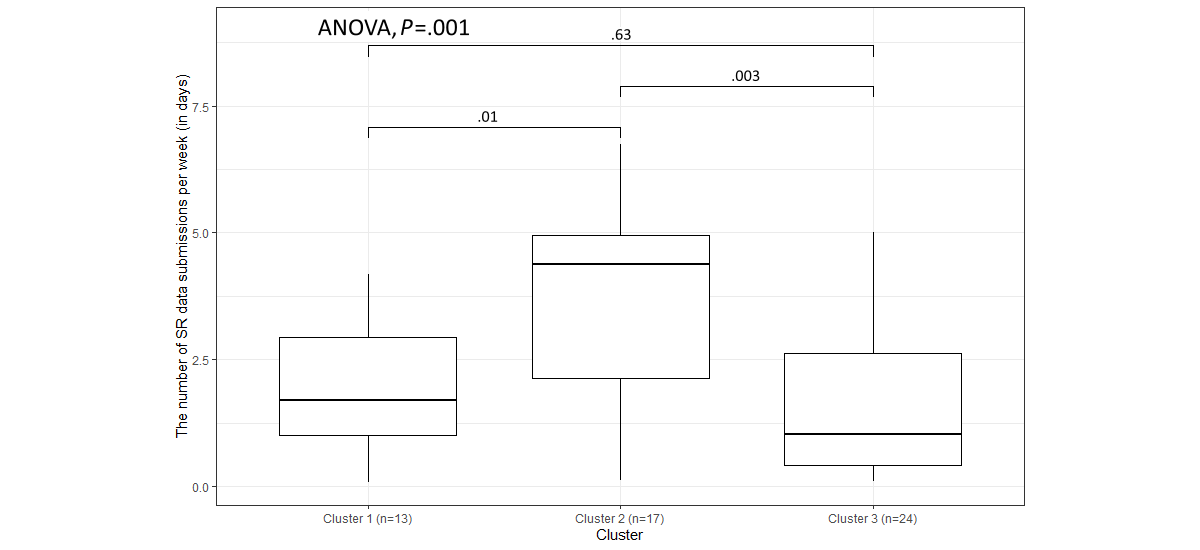
The variation in weekly submissions (days) for self-report (SR) data within the 3 clusters.

As Figure 12 shows, the distribution of SR data submissions is different, with the *P* value of the 1-way ANOVA being .001. In Figure 12, the median of cluster 2 is higher than the medians of the other 2 clusters, and compared with other variables, but unlike other parameters, cluster 2 also has some participants who had very low SR data submission rates (close to 0). SR data is the only parameter where cluster 1 has a higher median than cluster 3.

**Table 3:** Weekly submissions (days) of parameters.

Parameter	Cluster 1 (n=13), mean (SD)	Cluster 2 (n=17)	Cluster 3 (n=24)
Blood pressure	2.5 (1.4)^a^	5.7 (0.7)^b^	2.9 (1.6)
Weight	1.2 (0.9)^a^	5.4 (0.8)^b^	1.8 (1.5)
Physical activity	5.2 (0.7)^a^	6.7 (0.5)^b^	6.5 (0.4)
Self-report data	1.9 (1.4)	3.7 (2.1)^b^	1.6 (1.4)^a^
Sleep	4.2 (1.3)^a^	6.5 (0.4)^b^	6.1 (0.6)

^a^Lowest submission rate across the clusters.

^b^Highest submission rate across the clusters.

### The Correlation Among the Weekly Submissions of Different Parameters

**Figure 13:**
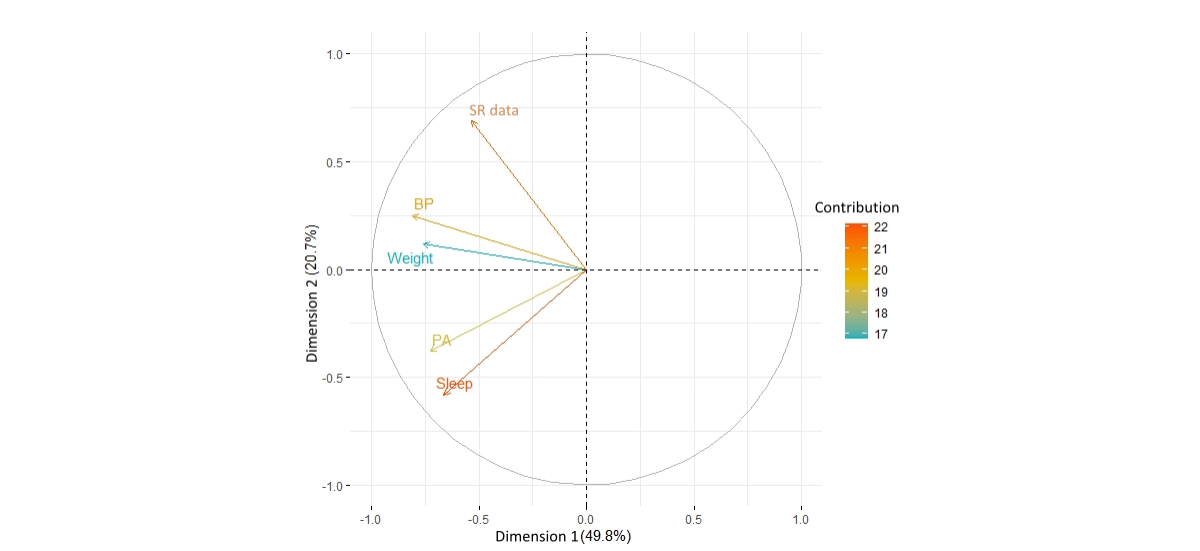
The principal component analysis for variables. BP: blood pressure; PA: physical activity; SR: self-report.

As seen in Figure 13, the arrows of BP and weight point to the same side of the plot, which shows a strong correlation. Likewise, PA and sleep also have a strong correlation. As noted previously, the strong correlation between PA and sleep is because the same device collected these 2 measurements, and participants only needed to sync the data once a day. By contrast, BP and weight were collected by 2 different devices but are strongly correlated. During interviews, many participants mentioned that their daily routine with the ProACT platform involved taking both BP and weight readings:

Usually in the morning when I get out of the bed, first, I go into the bathroom, wash my hands and come back, then weigh myself, do my blood pressure, do my bloods.

P008

I now have a routine that I let the system read my watch first thing, then I do my blood pressure thing and then I do the weight.

P015

As I said, it’s keeping me in line with my, when I dip my finger, my weight, my blood pressure.

P040

I use it in the morning and at night for putting in the details of blood pressure in the morning and then the blood glucose at night. Yes, there’s nothing else, is there? Oh, every morning the [weight] scales.

P058

By contrast, as shown in Figure 13, SR data have a weak correlation with other parameters, for reasons noted earlier.

### Parameter Variation Over Time

**Figure 14:**
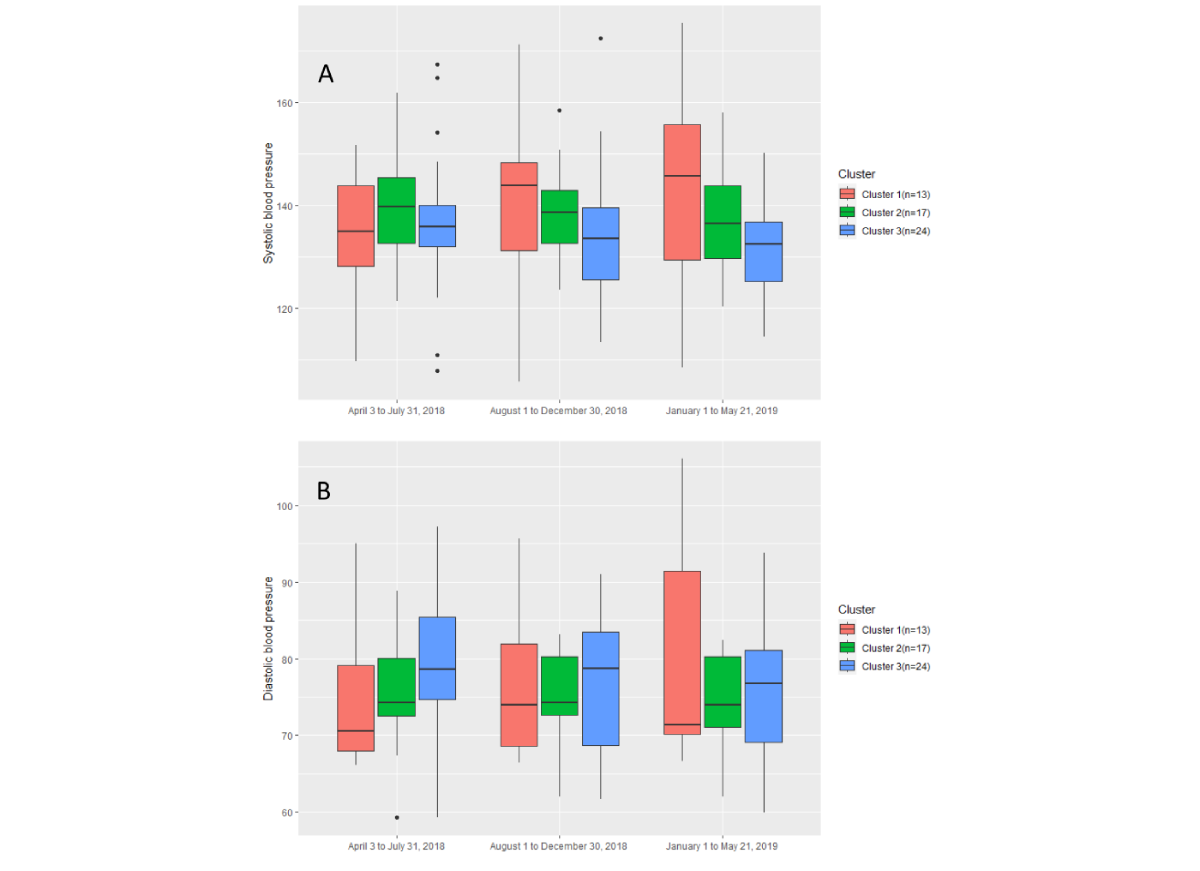
(A) The variation in systolic blood pressure in the 3 clusters among different time periods of the trial. (B) The variation in diastolic blood pressure in the 3 clusters among different time periods.

Analysis was conducted to determine any differences among the clusters in terms of symptom and well-being parameter changes over the course of the trial. Table 4 provides a description of each cluster in this regard. As Figure 14 shows, the box plot of cluster 2 is comparatively short in every time period of the trial, and the medians of cluster 2 and cluster 3 are more stable than the median of cluster 1. In addition, the median of cluster 1 is increasing over time, whereas the medians of cluster 2 and cluster 3 are decreasing and within the normal systolic BP of older adults [49] (Figure 14). As can be seen in Table 5, cluster 2 has a *P* value of .51 for systolic BP and a *P* value of .52 for diastolic BP, which are higher than the *P* values of cluster 1 (*P*=.19 and *P*=.16, respectively) and cluster 3 (*P*=.27 and *P*=.35, respectively). Therefore, participants in cluster 2, as highly engaged users, have more stable B*P* values than those in the other 2 clusters. By contrast, participants in cluster 1, as the least engaged users, have the most unstable B*P* values.

**Figure 15:**
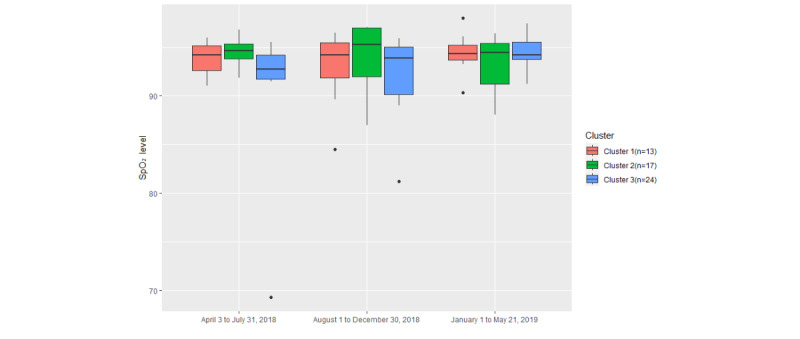
The variation in peripheral oxygen saturation (SpO2) levels in the 3 clusters among different time periods.

As seen in Figure 15, the median of cluster 2 is relatively higher than the medians of the other 2 clusters. The median of cluster 3 is increasing over time. In the second and third time periods of the trial, the box plot of cluster 1 is comparatively short. Normal SpO_2_ levels are between 95% and 100%, but older adults may have SpO_2_ levels closer to 95% [50]. In addition, for patients with COPD, SpO_2_ levels range between 88% and 92% [51]. In this case, there is not much difference in terms of SpO_2_ levels, and most of the SpO_2_ levels are between 90% and 95% in this study. However, the SpO_2_ levels of cluster 1 and cluster 2 were maintained at a relatively high level during the trial. As for cluster 3, the SpO_2_ levels were comparatively low but relatively the same as those in the other 2 clusters in the later period of the trial. Therefore, the SpO_2_ levels of cluster 3 (*P*=.25) are relatively unstable compared with those of cluster 1 (*P*=.66) and cluster 2 (*P*=.59). As such, there is little correlation between SpO_2_ levels and engagement with digital health monitoring.

**Figure 16:**
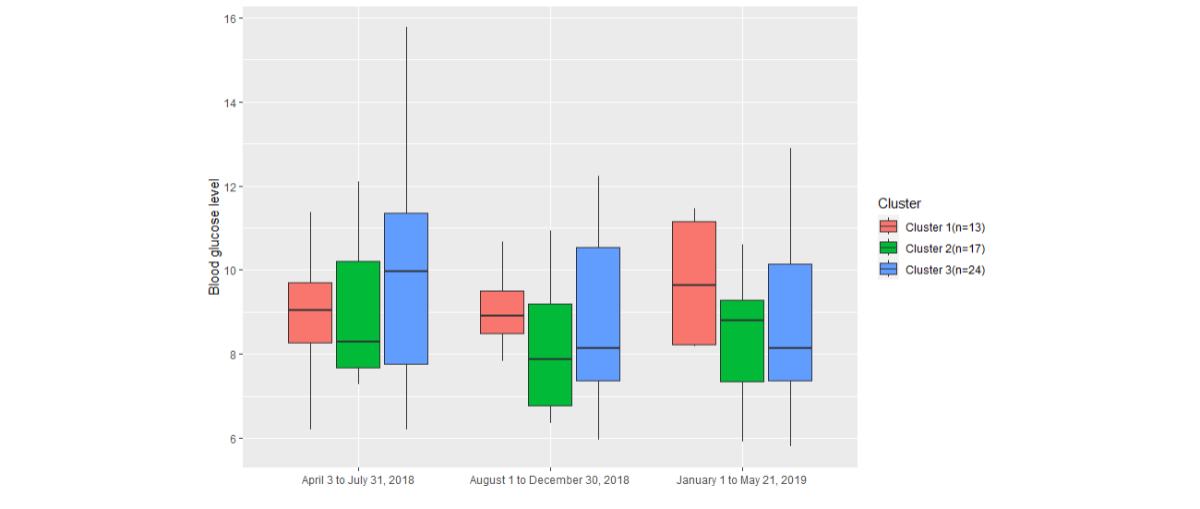
The variation in blood glucose levels in the 3 clusters among different time periods.

In relation to BG, Figure 16 shows that the box plot of cluster 2 is relatively lower than the box plots of the other 2 clusters in the second and third time periods. Moreover, the medians of cluster 2 and cluster 3 are lower than those of cluster 1 in the second and third time periods. The BG levels in cluster 2 and cluster 3 decreased at later periods of the trial compared with the beginning of the trial, but those in cluster 1 increased. Cluster 3 (*P*=.25), as the typical user group, had more significant change than cluster 1 (*P*=.50) and cluster 2 (*P*=.41). Overall, participants with a higher engagement rate had better BG control.

**Figure 17:**
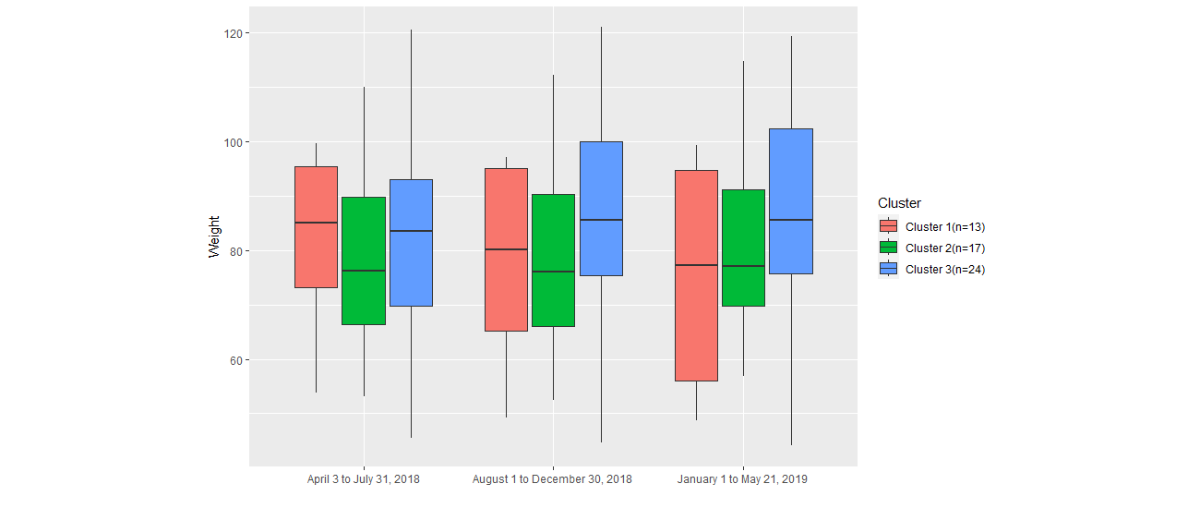
The variation in weight in the 3 clusters among different time periods.

In relation to weight, Figure 17 shows that the box plot of cluster 2 is lower than the box plots of the other 2 clusters and comparatively short. As Table 5 shows, the *P* value of cluster 2 weight data is .72, which is higher than the *P* values of cluster 1 (.47) and cluster 3 (.61). Therefore, participants in cluster 2 had a relatively stable weight during the trial. In addition, as seen in Figure 17, the median weight of cluster 1 participants is decreasing, whereas that of cluster 3 participants is increasing. It is well known that there are many factors that can influence body weight, such as PA, diet, environmental factors, and so on. [52]. In this case, engagement with digital health and well-being monitoring may help control weight but the impact is not significant.

**Figure 18:**
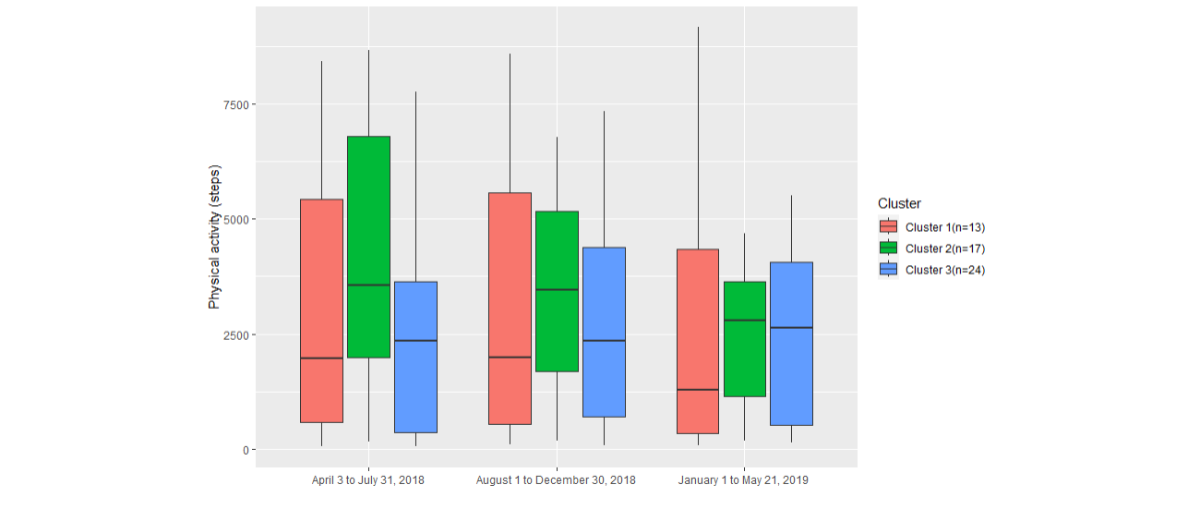
The variation in physical activity in the 3 clusters among different time periods.

As Table 5 shows, the *P* value of cluster 2 PA (.049) is lower than .05, which means that there are significant differences among the 3 time slots in cluster 2. However, the median of cluster 2 PA, as seen in Figure 18, is still higher than the medians of the other 2 clusters. In cluster 2, approximately 50% of daily PA (steps) consists of >2500 steps. Overall, participants with a higher engagement rate also had a higher level of PA.

**Table 4:** The description of each cluster.

Cluster	Description	Label
Cluster 1	In cluster 1, each feature and submission rate are lower than those in the other 2 clusters, and cluster 1 has the least participants among the clusters. Typically, users have increasing systolic BP^a^ over time, decreasing weight over time, and unstable BG^b^ levels over time.	Least engaged user
Cluster 2	In cluster 2, every parameter’s submission rate is higher than that in the other 2 clusters, the average submission rate is high, and the SDs of the submission rates are low except in the case of SR^c^ data. Typically, users have stable BP over time, which is also within the recommended thresholds.	Highly engaged user
Cluster 3	In cluster 3, the submission rates for PA^d^ and sleep are high, and the submission rates of the other 3 parameters are lower than those of cluster 2. However, cluster 3, which includes 44% (24/54) of the participants, is the largest cluster. The users’ systolic BP usually decreases over time.	Typical user

^a^BP: blood pressure.

^b^BG: blood glucose.

^c^SR: self-report.

^d^PA: physical activity.

**Table 5:** The *P* value of each cluster among all time slots by 1-way ANOVA.

Cluster and parameters		*P* value
**Cluster 1**		
	Systolic BP^a^	.19
	Diastolic BP	.16
	SpO_2_^b^	.66
	BG^c^	.50
	Weight	.47
	PA^d^	.68
**Cluster 2**		
	Systolic BP	.51
	Diastolic BP	.52
	SpO_2_	.59
	BG	.41
	Weight	.72
	PA	.049
**Cluster 3**		
	Systolic BP	.27
	Diastolic BP	.35
	SpO_2_	.25
	BG	.22
	Weight	.61
	PA	.86

^a^BP: blood pressure.

^b^SpO_2_: peripheral oxygen saturation.

^c^BG: blood glucose.

^d^PA: physical activity.

## Discussion

### Principal Findings

Digital health technologies hold great promise to help older adults with multimorbidity to improve health management and health outcomes. However, such benefits can only be realized if users engage with the technology. The aim of this study was to explore the engagement patterns of older adults with multimorbidity with digital self-management by using data mining to analyze users’ weekly submission data. Three clusters were identified: cluster 1 (the least engaged user group), cluster 2 (the highly engaged user group), and cluster 3 (the typical user group). The subsequent analysis focused on how the clusters differ in terms of participant characteristics, patterns of engagement, and stabilization of health condition symptoms and well-being parameters over time, as well as how engagement rates with the different devices correlate with each other.

The key findings from the study are as follows:

- There is no significant difference in participants’ characteristics among the clusters in general. The highly engaged group had the lowest average age (Table 4), and there was no significant difference with regard to sex and health conditions among these clusters. The least engaged user group had fewer male participants and participants with diabetes.
- There are 3 main factors influencing the correlations among the submission rates of different parameters. The first concerns whether the same device was used to submit the parameters, the second concerns the number of manual operations required to submit the parameter, and the third concerns the daily routine of the participants.
- Increased engagement with devices may improve the participants’ health and well-being outcomes (eg, symptoms and PA levels). However, the difference between the highly engaged user group and the typical user group was relatively minimal compared with the difference between the highly engaged user group and the least engaged user group.

Each of these findings is discussed in further detail in the following subsections.

Although the findings presented in this paper focus on engagement based on the ProACT trial participants’ use data, the interviews that were carried out as part of the trial identified additional potential factors of engagement. As reported in the study by Doyle et al [44], participants spoke about how they used the data to support their self-management (eg, taking action based on their data) and experienced various benefits, including increased knowledge of their health conditions and well-being, symptom optimization, reductions in weight, increased PA, and increased confidence to participate in certain activities as a result of health improvements. The peace of mind and encouragement provided by the clinical triage service as well as the technical support available were also identified during the interviews as potential factors positively impacting engagement [44]. In addition, the platform was found to be usable, and it imposed minimal burden on participants (Table 1). These findings supplement the quantitative findings presented in this paper.

### Age, Sex, Health Condition Types, and Engagement

In this study, the difference in engagement with health care technologies between the sex was not significant. Of the 23 female participants, 6 (26%) were part of the least engaged user group compared with 7 (23%) of the 31 male participants. Moreover, there were lower proportions of female participants in the highly engaged user group (7/23, 30%) and typical user group (10/23, 43%) compared with male participants (10/31, 32% and 14/31, 45%, respectively). Other research has found that engagement with mobile health technology for BP monitoring was independent of sex [53]. However, there are also some studies that show that female participants are more likely to engage with digital mental health care interventions [54,55]. Therefore, sex cannot be considered as a separate criterion when comparing engagement with health care technologies, and it was not found to have significant impact on engagement in this study. Regarding age, many studies have shown that younger people are more likely to use health care technologies than older adults [56,57]. Although all participants in our study are older adults, the highly engaged user group is the youngest group. However, there was no significant difference in age among the clusters, with some of the oldest users being part of cluster 3, the typical user cluster. Similarly, the health conditions of a participant did not significantly impact their level of engagement. Other research [53] found that participants who were highly engaged with health monitoring had higher rates of hypertension, chronic kidney disease, and hypercholesterolemia than those with lower engagement levels. Our findings indicate that the highly engaged user group had a higher proportion of participants with diabetes, and the least engaged user group had a higher proportion of participants with COPD. Further research is needed to understand why there might be differences in engagement depending on health conditions. In our study, participants with COPD also self-reported on certain symptoms, such as breathlessness, chest tightness, and sputum amount and color. Although engagement with specific questions was not explored, participants in cluster 1, the least engaged user group, self-reported more frequently than those in cluster 3, the typical user group. Our findings also indicate that participants monitoring BG level and BP experienced better symptom stabilization over time than those monitoring SpO_2_ level. It has been noted that the expected benefits of technology (eg, increased safety and usefulness) and need for technology (eg, subjective health status and perception of need) are 2 important factors that can influence the acceptance and use of technology by older adults [58]. It is also well understood that engaging in monitoring BG level can help people with diabetes to better self-manage and make decisions about diet, exercise, and medication [59].

### Factors Influencing Engagement

Many research studies use *P* values to show the level of similarity or difference among clusters [60-63]. For most of the engagement outcomes in this study, all clusters significantly differed, with 1-way ANOVA *P*<.001, with the exception being SR data (*P*=.001). In addition, the 2-tailed *t* test *P* values showed that cluster 2 was significantly different from cluster 1 and cluster 3 in BP and weight data submission rates, whereas cluster 1 was significantly different from cluster 2 and cluster 3 in PA and sleep data submission rates. As for SR data submission rates, all 3 two-tailed *t* tests had *P* values >.001, meaning that there were no significant differences between any 2 of these clusters. Therefore, all 5 parameters used for clustering were separated into 3 groups based on the correlations of submission rates: 1 for BP and weight, 1 for PA and sleep, and 1 for SR data. PA and sleep data submission rates have a strong correlation because participants used the same device to record daily PA and sleeping conditions. SR data submission rates have a weak correlation with other parameters’ submission rates. Our previous research found that user retention in terms of submitting SR data was poorer than user retention in terms of using digital health devices, possibly because more manual operations are involved in the submission of SR data than other parameters or because the same questions were asked regularly, as noted by P027 in the *Participant Engagement Outcomes* subsection [64].

Other research that analyzed engagement with a diabetes support app found that user engagement was lower when more manual data entry was required [65]. In contrast to the other 2 groups of parameters, BP and weight data are collected using different devices. Whereas measuring BP requires using a BP monitor and manually synchronizing the data, measuring weight simply requires standing on the weight scale, and the data are automatically synchronized. Therefore, the manual operations involved in submitting BP and weight data are slightly different. However, the results showed a strong correlation between BP and weight because many participants preferred to measure both BP and weight together and incorporate taking these measurements into their daily routines. Research has indicated that if the use of a health care device becomes a regular routine, then participants will use it without consciously thinking about it [66]. Likewise, Yuan et al [67] note that integrating health apps into people’s daily activities and forming regular habits can increase people’s willingness to continue using the apps. However, participants using health care technology for long periods of time might become less receptive to exploring the system compared with using it based on the established methods to which they are accustomed [68]. In this study, many participants bundled their BP measurement with their weight measurement during their morning routine. Therefore, the engagement rates of interacting with these 2 devices were enhanced by each other. Future work could explore how to integrate additional measurements, such as monitoring SpO_2_ level as well as self-reporting into this routine (eg, through prompting the user to submit these parameters while they are engaging with monitoring other parameters, such as BP and weight).

### Relationship Between Engagement and Health and Well-Being Outcomes

Our third finding indicates that higher levels of engagement with digital health monitoring may result in better outcomes, such as symptom stabilization and increased PA levels. Milani et al [69] found that digital health care interventions can help people achieve BP control and improve hypertension control compared with usual care. In their study, users in the digital intervention group took an average of 4.2 readings a week. Compared with our study, this rate is lower than that of cluster 2 (5.7), the highly engaged user group, but higher than cluster 1 (2.5) and cluster 3 (2.9) rates. In our study, participants with a higher engagement rate experienced more stable BP, and for the majority of these participants (34/41, 83%), levels were maintained within the recommended thresholds of 140/90 mm Hg [70]. Many studies have shown that as engagement in digital diabetes interventions increases, patients will experience greater reductions in BG level compared with those with lower engagement [71,72]. However, in our study, BG levels in both the highly engaged user group (cluster 2) and the least engaged user group (cluster 1) increased in the later stages of the trial. Only the BG levels of the typical user group (cluster 3) decreased over time, which could be because the cluster 3 participants performed more PA in the later stages of the trial than during other time periods, as Figure 18 shows. Cluster 2, the highly engaged user group, maintained a relatively high level of PA during the trial period, although it continued to decline throughout the trial. Other research shows that more PA can also lead to better weight control and management [73,74], which could be 1 of the reasons why cluster 2 participants maintained their weight.

### Limitations

There are some limitations to the research presented in this paper. First, although the sample size (n=60) was relatively large for a digital health study, the sample sizes for some parameters were small because not all participants monitored all parameters. Second, the participants were clustered based on weekly submissions of parameters only. If more features were included in clustering, such as submission intervals, participants could be grouped differently. It should also be pointed out that correlation is not a causality with respect to analyzing engagement rates with outcomes.

### Conclusions

This study presents findings after the clustering of a data set that was generated from a longitudinal study of older adults using a digital health technology platform (ProACT) to self-manage multiple chronic health conditions. The highly engaged user group cluster (includes 17/54, 31% of users) had the lowest average age and highest frequency of submissions for every parameter. Engagement with digital health care technologies may also influence health and well-being outcomes (eg, symptoms and PA levels). The least engaged user group in our study had relatively poorer outcomes. However, the difference between the outcomes of the highly engaged user group and those of the typical user group is relatively small. There are 3 possible reasons for the correlations between the submission rates of parameters and devices. First, if 2 parameters are collected by the same device, they usually have a strong correlation, and users will engage with both equally. Second, the devices that involve fewer steps and parameters with less manual data entry will have a weak correlation with those devices that require more manual operations and data entry. Finally, participants’ daily routines also influence the correlations among devices; for example, in this study, many participants had developed a daily routine to weigh themselves after measuring their BP, which led to a strong correlation between BP and weight data submission rates. Future work should explore how to integrate the monitoring of additional parameters into a user’s routine and whether additional characteristics, such as the severity of disease or technical proficiency, impact engagement.

## Abbreviations

BG: blood glucose
BP: blood pressure
CABIE-SIMS: Context-Aware Broker and Inference Engine–Subject Information Management System
COPD: chronic obstructive pulmonary disease
HD: heart disease
HF: heart failure
PC: principal component
PCA: principal component analysis
ProACT: Integrated Technology Systems for Proactive Patient Centred Care
SpO2: peripheral oxygen saturation
SR: self-report
